# Selecting Diversified Compounds to Build a Tangible Library for Biological and Biochemical Assays

**DOI:** 10.3390/molecules15075031

**Published:** 2010-07-23

**Authors:** Qiong Gu, Jun Xu, Lianquan Gu

**Affiliations:** Research Center for Drug Discovery, School of Pharmaceutical Sciences, Sun Yat-Sen University, 132 East Circle at University City, Guangzhou, 510006, China

**Keywords:** diversity, cheminformatics, chemical library, compound selection

## Abstract

The quality of diverse compound selection mainly depends on cluster algorithms, descriptors, the combinations of the descriptors, and similarity metrics. The Jarvis-Patrick algorithm, MDL search keys, and Daylight fingerprints are a well accepted algorithm and structure descriptors for compound library diversity analysis. Based upon our 288 experiments on selecting compounds from various descriptor combinations, we have found (1) hybrid Daylight and MDL structural descriptors for diversity analyses can produce worse results; (2) selections based purely on 2,048-bit Daylight fingerprints yield better results than the ones based purely on MDL 166-bit search keys; (3) when Daylight fingerprints and MDL search keys are combined, it is better to compute the similarities independently, then to take the smaller value for the outcome. This will yield better average separation of clusters; (4) regarding the consistency of different clustering approaches, the Daylight fingerprints based clustering is more consistent with the SCA approach than it does with the MDL search keys based approach; (5) The MDL search keys based selection approach tends to select a greater number of compounds from larger clusters. As the Daylight fingerprint is folded two and three times, respectively, information is lost, and this approach tends to select a greater number of compounds from larger clusters as well. These results have not been reported before to our knowledge.

## 1. Introduction

A tangible compound repository is a prime resource for an integrated drug discovery platform. The Guangdong Small Molecule Tangible Library (GSMTL) project was initiated to accelerate the drug discovery process. The goal of this project is to publicly provide diversified small molecules for biological and biochemical assays. Millions of compounds are commercially available in the world. Limited budgets and facilities require us to select diverse and lead-like compounds for a tangible library repository.

The compound acquisition process consists of the following steps:

(1) collecting compound libraries (virtual compounds) from vendors worldwide,
(2) filtering unwanted compounds from the virtual libraries for toxiphores, aggregation false positives, protein reactive false positives, promiscuous inhibitors, frequent hitters, and warhead agents [1,2,3,4,5,6,7,8,9,10]
(3) filtering unwanted compounds based upon criteria, such as, purity > 90% , quantity > 10 mg, MW < 500 (if it is not considered to be a natural product), predicted solubility > 20 µg/mL, and no undesirable functional groups,
(4) clustering the remaining virtual compounds, and selecting representative compounds for each structural group,
(5) initially, picking a number of fragment-like, lead-like, and natural-product-like (TCM-like) compounds [11,12,13]
(6) picking additional compounds, and adding to the previous collection to maximize the structural diversity of the compound repository.

Many clustering methods can be used to select structurally diverse compounds. The results rely on: (1) structural descriptors, (2) ways to treat the descriptors, (3) similarity metrics, and (3) a diversity algorithm. The question is: how does one select descriptor sets? People tend to believe in a consensus approach. It is thought that combining the MDL search keys (SK) and Daylight fingerprints (DF) may produce better results.

**Figure 1:**
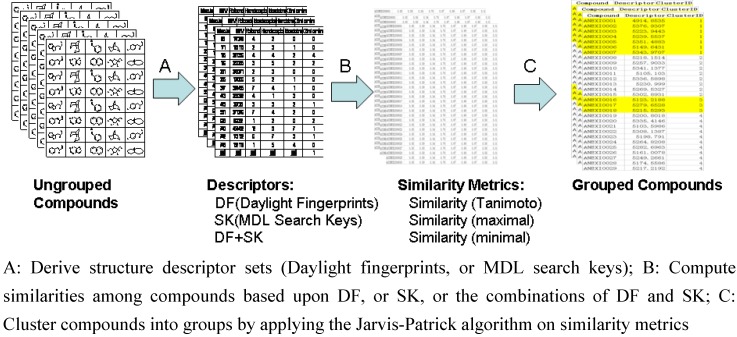
Computational process for grouping compounds in a library.

In order to evenly sample the space of the chemical diversity of small molecules for the GSMTL project, the Jarvis-Patrick algorithm [14,15,16] was used to cluster virtual compounds into groups, then select representative compounds from each group. Two types of structural descriptor sets, the Daylight fingerprints and the MDL search keys, were used for the cluster algorithm. Figure 1 shows the computational process of grouping compounds for compound acquisition.

As shown in Figure 1, there are many options for computing similarity metrics at step B. The similarity matrix can be calculated from (1) MDL 166-bit search keys, (2) 2,048-bit Daylight fingerprints, or (3) the combinations of both. It was hypothesized that the descriptor sets from combined DF and SK may produce better results.

The information content of 2,048-bit Daylight fingerprints is not very high because many bits are unset. In order to improve the computational performance, the 2,048-bit Daylight fingerprints are usually folded to 1,024, 512, or 256 bits. MDL published 960-bit search keys as well, but Durant and colleagues concluded that increasing keyset size had little effect on overall performance. Thus, it was decided that only 166-bit search keys were to be used in this study [17].

When two types of keys are combined, three similarity metrics are available:

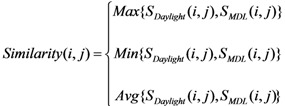
(1)
where S*_Daylight_*(i,j) represents the similarity of structure*_i_* and structure*_j_* calculated from pure Daylight fingerprints; S*_MDL_*(i,j) represents the similarity of structure*_i_* and structure*_j_* calculated from pure MDL search keys. Similarity (i, j) is the final similarity value between structure*_i_* and structure*_j_*. Max{x, y}, Min{x, y}, or Avg{x, y} stand for taking the greater value, smaller value, or the average value between x and y, respectively.

Therefore, the options for structural descriptor sets in a similarity metric calculation can be:

D1: 2,048-bit Daylight fingerprints (DF),
D2: 166-bit MDL search keys (SK),
D3: Max{DF, SK},
D4: Min{DF, SK},
D5: Avg{DF, SK},
D6: 2,214-bit combined keys (2048+166 bits),
D7: 1,190-bit combined keys (1024+166 bits),
D8: 678-bit combined keys (512+166 bits),
D9: 422-bit combined keys (256+166 bits).

**Figure 2:**
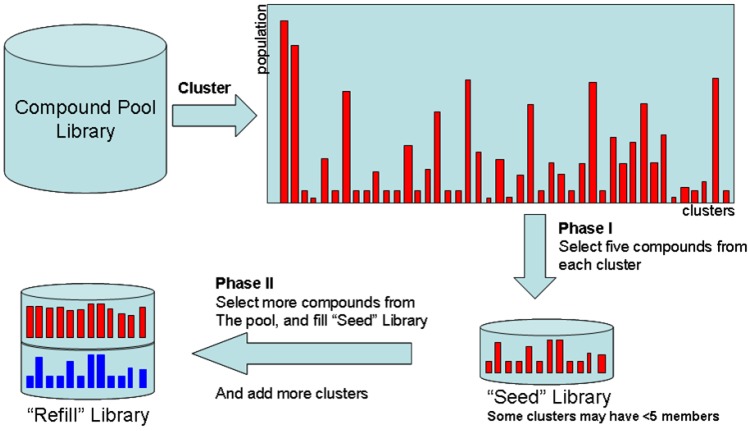
The process of diversity-based compound acquisition.

The compound selection process is divided into phase I and phase II, as shown in Figure 2. Phase I selects *n* compounds from a compound pool based upon diversity; these selected compounds are denoted as the “Seed” library. Phase II adds more compounds to the “Seed” library. The rules for adding new compounds are (1) more compounds for the cluster groups that do not have enough members (usually a compound cluster should have at least five members), (2) compounds belonging to new clusters are sent to “Seed” libraries. 

The goal of this study is to figure out a decent computational protocol to select diversified compounds with which to build a tangible library for biological and biochemical assays. In order to validate the Jarvis-Patrick algorithm based compound selection protocols, the structure/scaffold based approach (SCA) [18], a non-descriptor-based method, was used as an objective validation tool.

In order to figure out the best descriptor sets and their combination for selecting tangible compounds for preliminary screens, selection techniques have been applied to the Maybridge virtual database. The protocols for the selection experiments are described in the Experimental Section.

## 2. Results and Discussion

### 2.1. The information content of descriptor sets

**Figure 3:**
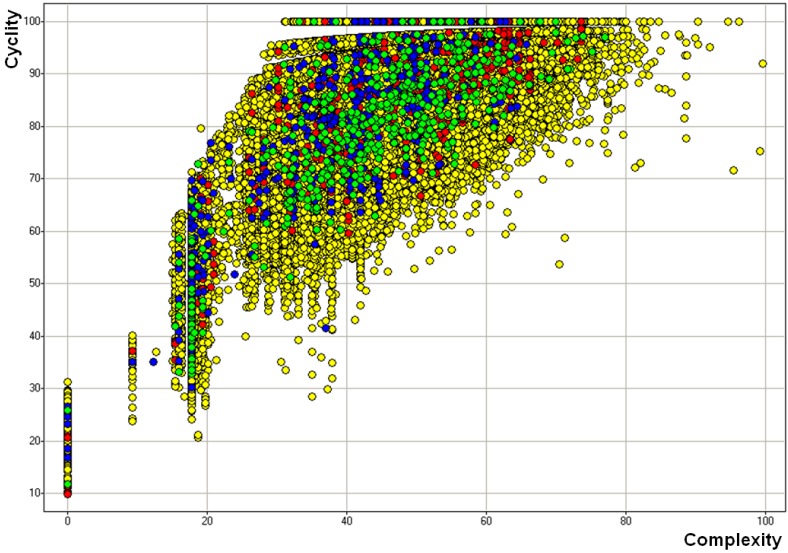
SCA clusters comparing the picking of compounds using pure Daylight (D1), in red, pure MDL (D2), in blue, and randomly, in green. The yellow dots represent the entire Maybridge diversity space. The compounds in a vertical line belong to the same cluster.

Descriptor sets are represented in binary bit-maps. The bit-maps are not set evenly; many bits are unset. The average bit-setting probabilities for bits have been calculated as shown in Figure 3. The average bit-setting probability represents information content for each descriptor. 256-bit Daylight descriptor sets have the highest bit-setting probability; however, it has higher average “bits on” entropy (more noise).

### 2.2. Classification Methods

The Jarvis-Patrick *k*NN approach [20] is a non-hierarchical clustering algorithm, which connects two structures if they share *k* common neighbors. If the similarity of two structures is greater than a given threshold, then they are neighbors. In order to validate the clustering results from different similarity metrics, we use the scaffold based classification approach (SCA) to measure the numbers of scaffolds found in the results from different similarity metrics.

The SCA method computes the complexity and cyclicity for each compound. The complexity represents the molecular size and the number of bonds, and the cyclicity represents the percentage of ring bonds over the all bonds in the molecule. The complexity and cyclicity are defined in reference [18].

A scatter plot of the complexity over the cyclicity, as calculated with SCA, can graphically show a clustering of compounds around a single scaffold, depicted along the vertical lines. The closer the vertical dots are to each other, the more similar the compounds are. Figure 3 shows all compounds in the Maybridge collection (yellow), and three 500-“Seed” libraries picked by pure Daylight (D1, red), pure MDL (D2, blue), and Random (R1_S, green). Compounds picked by the random method are more evenly distributed in the diversity space than the other methods’, primarily because the random selection did not attempt to select compounds as clusters of 5 in size. The pure Daylight (D1) and pure MDL (D2) approaches show better grouping than the random selection. All three methods seem to pick compounds evenly across the full diversity space.

**Figure 4:**
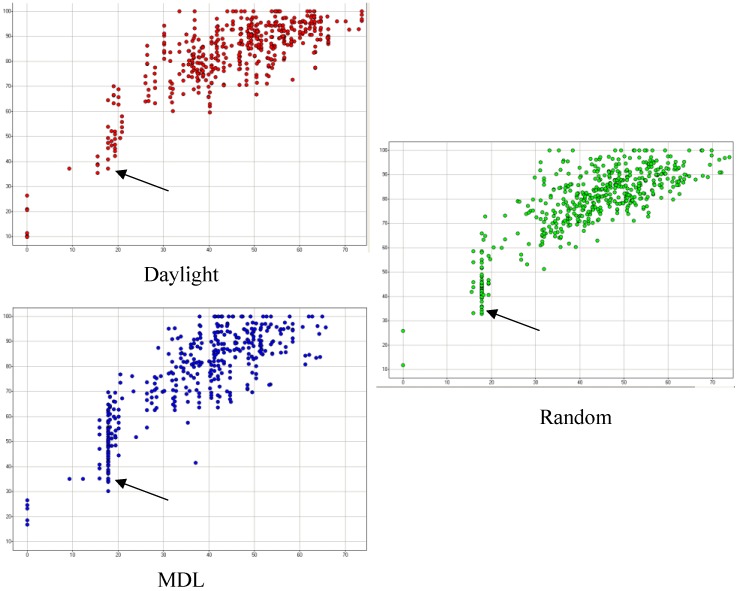
Selected compounds analyzed by SCA, separated by pure Daylight (D1), pure MDL (D2), and R1_S.

Figure 4 shows a simplified depiction of Figure 3 where D1, D2, and R1_S selections are separated. One can see that the D1 selection has good separation of small clusters, which upon closer inspection, correlate closely with SCA vertical lines. Although D2 selection also shows good separation of small clusters, upon closer inspection, mono-substituted benzene rings are over populated on that SCA cluster (vertical line indicated by arrows in Figure 4). Since the Maybridge database has a large benzene derivative family, the random selection would be expected to select many of them. Also, the random selection has little vertical clustering, except for compounds with mono-substituted benzene rings.

It is also observed that both the MDL-based approach and random picking tend to pick more benzene derivates (marked by arrows in Figure 4). Since the Maybridge compounds contain a big benzene derivative family, the random selection is expected to select many of them. The Daylight fingerprints, with their larger size and higher information entropy, is expected to group the benzene derivative family together and only select one or two clusters out of it.

**Figure 5:**
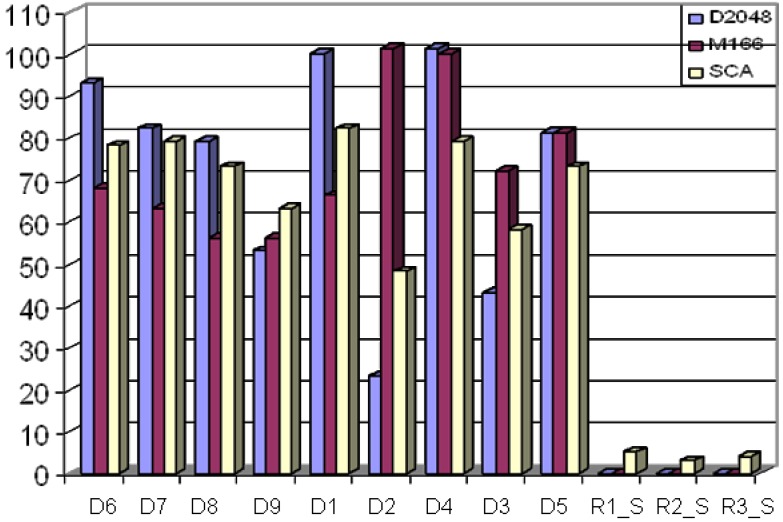
The Y-axis represents the number of valid compound clusters. The X-axis represents various clustering approaches with different similarity metrics, and random selections for comparisons. The nine Seed Libraries were selected based on nine types of descriptors (D1~D9), other three Seed Libraries (R1_S ~ R3_S) were selected randomly. The diversities of all twelve libraries are measured by means of three clustering methods (D2048, M168, and SCA). D4 scores are the best when all three analyses are considered. R1_S, R2_S, and R3_S, as expected, have very poor diversity (lowest numbers of the valid clusters).

In order to contrast the different selection methods, twelve different selections based on D1-D9, R1_S, R2_S, and R3_S were performed for the phase I (R1_S, R2_S, and R3_S are random selections). Then, three different clustering methods were used to measure the number of “Valid Clusters” (clusters have at least five members) in each selection set. The three clustering methods are: D1, D2, and SCA. The number of compounds in a cluster must qualify as a “Valid Cluster”. Figure 5 depicts the number of “Valid Clusters” across each of the nine selection methods, plus three random selections in the Seed Library of about 500 compounds. Figure 5 shows that, although D2 selection has the highest score for D2 analysis and that, D1 selection has the highest scores for D1 and SCA analyses, it is only D4 that consistently has high scores for all three clustering methods. In the Seed Library, D4 ranks 1^st^ for D1, 1^st^ for D2, and ties for 2^nd^ for SCA when discounting the trivial winner of D2 selection with D2 analysis. It should be noted that in almost all cases, the best method for a given analysis is the method that was used for selection. For example, D3 analysis ranks D3 selection 1^st^, or D9 analysis ranks D9 analysis 1^st^. These “trivial” winners are ignored, since it is the descriptor that can rank high consistently across multiple methods of analysis that is desired.

Figure 7 and Figure 8 show very similar graphs of the “Refill” and R1_S “Refill” selections. When comparing the two figures against each other, one can see that the primary difference is that the R1_S graphs have less “Valid Clusters” across the board. This is expected, since the R1_S is a randomly selected start set, which has little chance to construct valid clusters (clusters with at least five members).

**Figure 6:**
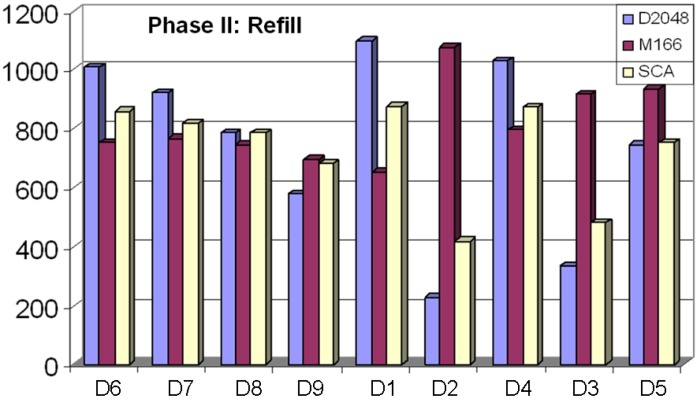
The “Refill” selection was done based on the Phase I selection, which contained 5,500 compounds (500 from “Seed”; 5,000 from “Refill”) across each descriptor used for the selection, using three separate clustering analyses. D4 scores the best when all three analyses are considered.

**Figure 7:**
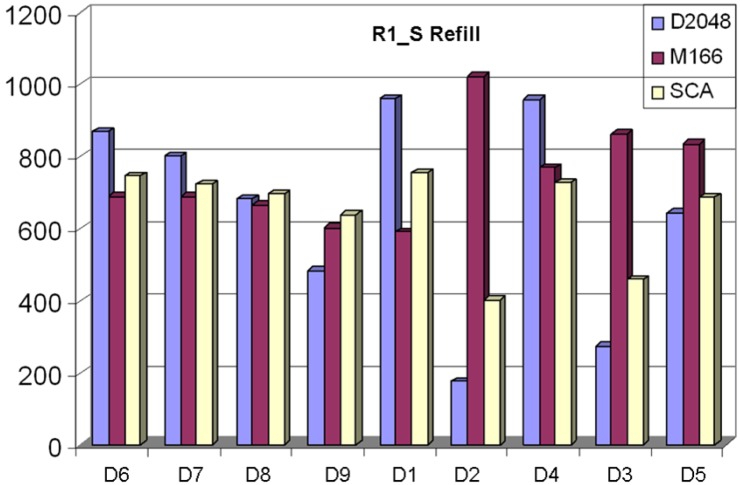
For comparison, the Refill selection was also done based on the R1_S (random start set) Seed library, which resulted in 5,500 compounds (500 from the R1_S “Seed” library, 5,000 from the “Refill”) across each descriptor used for the selection, using three separate clustering analyses. D4 scores the best when all three analyses are considered.

When comparing the selections for descriptor sets, D4 ranks the highest over all three clustering analyses. In Figure 6, D4 ranks 1^st^ for D1, 2^nd^ for SCA, and 3^rd^ for D2. In Figure 7, D4 ranks 1^st^ for D1, 2^nd^ for D2, and 3^rd^ for SCA. Also of note in these two figures is the widening gap between selection based on D2 and analysis based on D1 and, to a lesser extent, SCA. As discussed above, the Maybridge database has a large number of mono-substituted benzene rings, which the MDL search keys tend to place in separate clusters, but the Daylight fingerprint and SCA analysis tend to place in the same cluster. The D3 and D5 descriptor sets tend to perform more like MDL search keys, primarily because D2 Tanimoto similarity is generally higher than the D1 Tanimoto similarity.

These figures also show that a selection based purely on Daylight fingerprints yield good results overall, though the selection suffers somewhat when analyzed by MDL search keys. A selection based purely on MDL search keys performs poorly in both Daylight and SCA analysis.

**Figure 8:**
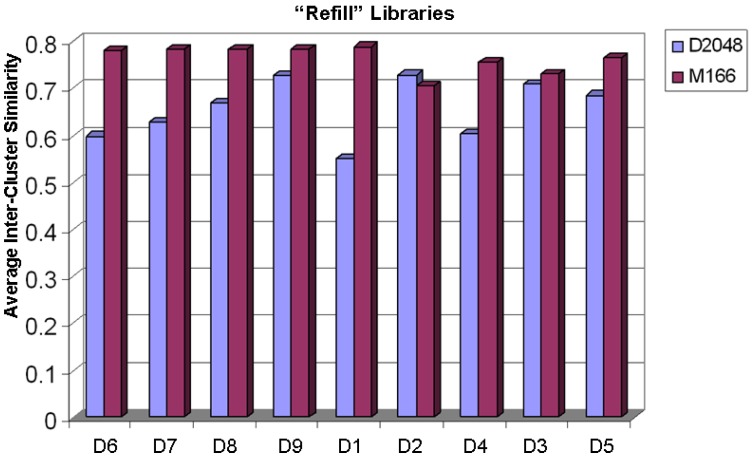
The refill libraries are validated by computing average inter-cluster similarity between the centroids of each cluster and its nearest cluster in the diversity space. The lower average inter-cluster similarity means high diversity. The measurements are based Daylight 2,048 bit-maps (D1) and MDL 166 search keys (D2).

To ensure that clusters are well separated after a selection, the similarity between cluster centroids is calculated for all pairs of clusters. Then, for each cluster, the closest, or maximally similar cluster centroid, is averaged over the entire collection to yield the Average Maximal Inter-cluster Similarity depicted in Figure 8. In this graph, a lower number indicates that cluster centroids are separated by a greater distance, which is desirable for a diverse collection. Discounting the trivial winners, D4 shows better separation of clusters with ranks of 2^nd^ for D1 and 2^nd^ for D2. 

**Figure 9:**
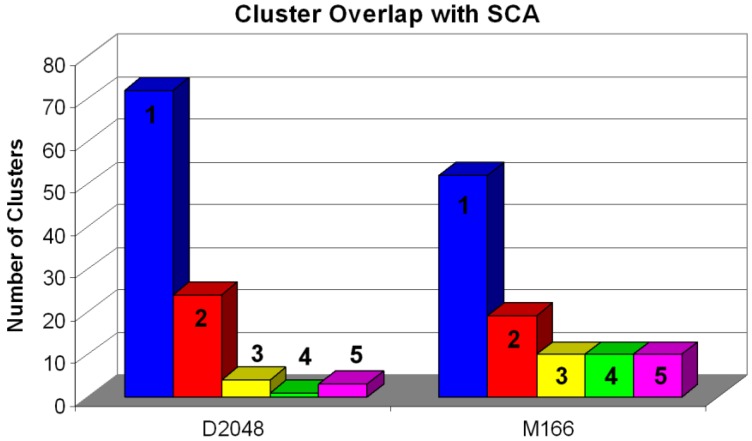
Seed libraries selected by D1 and D2 are validated by the SCA approach. The blue bar depicts the number of selected clusters, which exactly overlap with SCA derived clusters. The red bar depicts the number of selected clusters, which overlap with exactly 2 SCA derived clusters; yellow, green, and purple represent 3, 4, and 5 overlapping SCA clusters, respectively.

In order to validate the consistency of different clustering approaches, we have compared the clusters in different clustering methods to see if the same group of compounds is classified in the same groups in other approaches. If the compounds are placed in the same cluster by at least two clustering approaches, the cluster will be marked with “1”, which means good clustering consistency. If the cluster of compounds is split into two SCA clusters, this cluster is marked with “2”, which means less clustering consistency, and so forth. Figure 9 shows the degree of overlap that pure Daylight (D1) and pure MDL search keys (D2) selected clusters have with Scaffold-based Cluster Analysis. The calculation is based on seed libraries. As shown in Figure 9, both D1 and D2 descriptor sets have a high level of agreement on which compounds are clustered together. The D1 clustering method has a much higher correlation with the SCA method than it does in the D2. For example, D1 clustering has about 70 clusters, which are consistent to the SCA clustering results; D2 clustering has only 50 clusters that are consistent with the SCA clustering results. The bigger ranking numbers mean less consistency. As we can tell in Figure 9, D1 has less large-ranked-number (>2) clusters than the one D2 has (yellow, green, and purple bars). The SCA validation concludes that D1 is a better clustering approach than D2.

**Figure 10:**
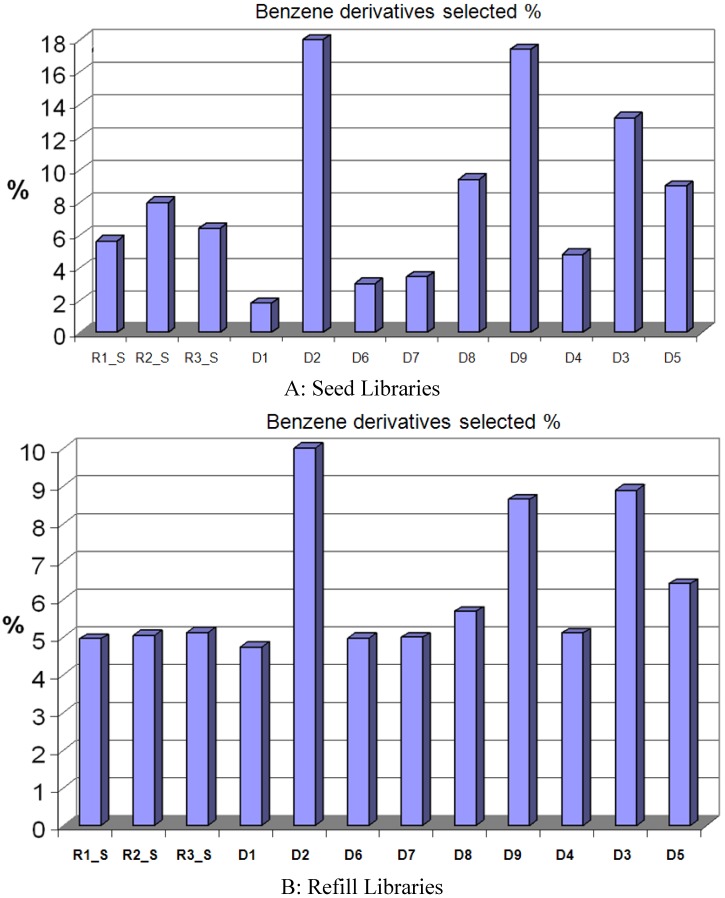
In comparison with the random selection approach, methods based on either Daylight bit-maps or MDL search keys have trends of oversampling compounds from the largest clusters. Since benzene derivates usually belong to the largest cluster, they are always oversampled.

The largest SCA clusters in all selected compound sets are always the benzene scaffold clusters. As shown in Figure 10, the MDL search keys based algorithm tends to select a greater number of compounds from the largest clusters. For the pure MDL method (D2), the “Seed” set contained 18% of the selected compounds from the benzene scaffold cluster, while the pure Daylight method (D1) selected only 1.8% of the compounds from this benzene scaffold cluster. With respect to all the combination methods, the D8 and D9 methods yielded the largest benzene scaffold clusters because, as the Daylight fingerprint was folded two and three times, respectively, information was lost, and also the D2 part of the fingerprint gained a greater overall weighting. Another combined method, D3, also had a very large benzene scaffold cluster, because it became biased towards the MDL search keys, which tended to have the higher similarity value. The same trends are found in the “Refill” compound sets as well.

**Figure 11:**
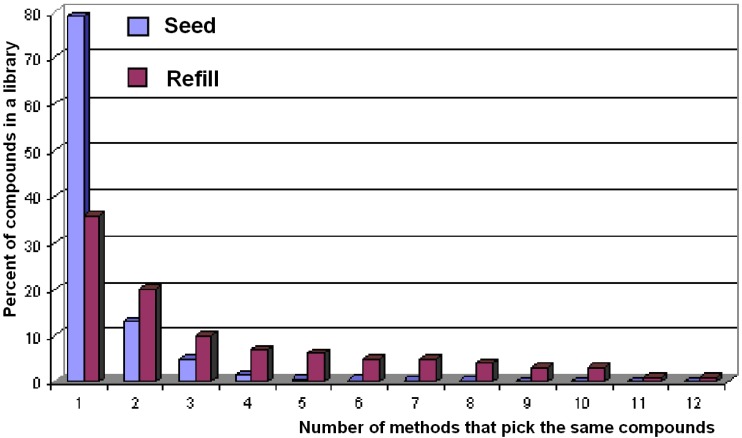
The distributions of compounds selected by 12 different methods. This figure shows that the compounds picked from different methods in the same compound pool have very little overlapping.

Compounds selected from different methods overlap very little, as shown in Figure 11. By taking the “Seed” libraries as examples, one can see that about 80% of the compounds are uniquely picked by one method, 13% of the compounds are picked by two methods, and no compound is picked by more than nine different methods. In the “Refill” libraries, only 8% of the compounds are picked by more than nine different methods.

It has been shown consistently throughout these experiments that the best method of combining two dissimilar fingerprint descriptor sets is to use the Minimum of the two similarities as the new combined similarity. When this new metric is used both for clustering and for selecting maximally dissimilar cluster centroids, the selected compounds will generally perform the best overall when analyzed with multiple different clustering methods.

## 3. Experimental

In order to mimic the compound selection process for the GSMTL project, the experiments are separated into two steps, “Start” and “Fill then Add”. The “Start” step selects 500 compounds that represent an inventory, which must be used as the basis upon which new compounds are added. The “Fill then Add” step selects 5,000 additional compounds that will fill any undersized clusters already present (<5), and then add new, diverse clusters to the existing collection.

The “Start” step consists of four separate collections. The first collection is generated by selecting diverse clusters, based purely on maximizing structural diversity, from the 60K compound test set. The other three collections are static sets of randomly selected compounds, denoted as R1_S, R2_S, and R3_S (random starts). The random sets are used to show that the selection algorithm can still achieve the target criterion given any set of existing seed compounds.

Each of the 9 descriptor sets (D1~D9) is tested to select four “Start” sets, each followed by a “Fill then Add” set. The resulting 72 selected compound sets will be analyzed by several diversity metrics:

(1) purely by Daylight fingerprints (D1)
(2) purely by MDL search keys (D2)
(3) Scaffold-based Classification Approach (SCA).

Given nine structural descriptor sets (D1~D9), three diversity analytical metrics (pure Daylight, pure MDL, and SCA), two steps of diversity selection, and four selection runs (one optimal, and three random), the total number of diversity experiments is 9 x 3 x 2 x 4 = 216.

2,048-bit Daylight fingerprints are derived from a chemical structure by looking over all the possible paths from 1 to 7 bonds along the structure [19]. The 166-bit MDL search keys are generated from predefined chemical functional groups (search keys). These substructures were derived from the most popular chemical groups based upon the chemists’ experience.

In order to make the experiments show statistically significant results, a Maybridge compound collection, which contains about sixty thousand unique and normalized structures, is used as the testing data set.

All the computational experiments have been accomplished on a Windows system. The descriptors have been calculated from MOE and in-house codes written in C language compiled by Visual C++ Version 6.0. The resulting data are available upon the requests.

## 4. Conclusions

These results are easy to justify when one considers that the critical step in clustering is the similarity metric, since it is expected that compounds have to be highly similar to each other in order to assemble into a given cluster. Of the nine descriptor sets tested, only the D4 guarantees a consensus between the Daylight and MDL based similarity calculations, where both metrics would agree when two compounds are similar. The D3 only ensures that one of the two descriptor sets reports high similarity, and even D5 holds the possibility that one descriptor scores very high, and the other scores, very low. Each of the concatenated fingerprint descriptor sets, mathematically, is just a variation of an average, but also has an additional problem of information loss in the folded fingerprint. This same justification can be applied to selection based on maximal dissimilarity, since this too relies on the critical step of similarity calculations, and must rely on a consensus between the two metrics.

In conclusion, a consensus metric that selects the minimum similarity over a set of descriptors has consistently shown the best performance when used to select a diverse set of clusters. This work has proved that the consensus approach is a good method. Additionally, because the Daylight 2,048 bit fingerprint encodes more than 10 times the amount of information than the 166 bit MDL search keys, the Daylight descriptors tend to yield a broader selection, where the clusters correlate better with a core scaffold.

When a molecule is described in an array (numbers or bit-maps); the components of the array should be orthogonal. However, neither the array of Daylight bit-maps nor the array of the MDL search keys can guarantee being orthogonal. A substructure represented by one bit can be the substructure of another substructure represented by a different bit in Daylight bit-maps or the MDL search keys. This means that some substructures are unintentionally biased. Consequently, the corresponding similarity metrics will lose objectivity. Increasing the width of bit-maps may reduce the concurrency, but the information content will be reduced, due to the increased number of empty bits (for example, increasing the length of the Daylight path from 3 to 7). Meanwhile, increasing the length of the Daylight path will increase computation cost without dramatic clustering enhancement. Experiments show that similarity metrics based on combined MDL search keys with Daylight bit-maps are worse than the ones using pure MDL keys or pure Daylight bit-maps. This is because the substructure concurrencies are increased. Hence, we do not recommend using physically hybrid structural descriptors for clustering or similarity metrics.

## References

[1] Rishton G.M. (1997). Reactive compounds and in vitro false positives in HTS. Drug Discov. Today.

[2] Rishton G.M. (2003). Nonleadlikeness and leadlikeness in biochemical screening. Drug Discov. Today.

[3] Roche O., Schneider P., Zuegge J., Guba W., Kansy M., Alanine A., BLleicher K., Danel F., Gutknecht E., Rogers-Evans M., Neidhart W., Stalder H., Dillon M., Sjögren E., Fotouhi N., Gillespie P., Goodnow R., Harris W., Jones P., Taniguchi M., Tsujii S., von der Saal W., Zimmermann G., Schneider G. (2002). Development of a virtual screening method for identification of ‘Frequent Hitters’ in compound libraries. J. Med. Chem..

[4] McGovern S.L., Helfand B.T., Feng B. (2003). A specific mechanism of nonspecific inhibition. J. Med. Chem..

[5] Seidler J., McGovern S.L., Doman T.N., Shoichet B.K. (2003). Identification and prediction of promiscuous aggregating inhibitors among known drugs. J. Med. Chem..

[6] McGovern S.L., Shoichet B.K. (2003). Kinase inhibitors: not just for kinases anymore. J. Med. Chem..

[7] Feng B.Y., Shoichet B.K. (2006). Synergy and antagonism of promiscuous inhibition in multiple-compound mixtures. J. Med. Chem..

[8] Shoichet B.K. (2006). Screening in a spirit haunted world. Drug Discov. Today.

[9] Feng B.Y., Simeonov A., Jadhav A., Babaoglu K., Inglese J., Shoichet B.K., Austin C.P. (2007). A high-throughput screen for aggregation-based inhibition in a large compound library. J. Med. Chem..

[10] Roche O., Schneider P., Zuegge J., Guba W., Kansy M., Alanine A., BLleicher K., Danel F., Gutknecht E., Rogers-Evans M., Neidhart W., Stalder H., Dillon M., Sjögren E., Fotouhi N., Gillespie P., Goodnow R., Harris W., Jones P., Taniguchi M., Tsujii S., von der Saal W., Zimmermann G., Schneider G. (2002). Development of a virtual screening method for identification of ‘Frequent Hitters’ in compound libraries. J. Med. Chem..

[11] Rishton G. M. (2008). Molecular diversity in the context of leadlikeness: compound properties that enable effective biochemical screening. Curr. Opin. Chem. Biol..

[12] Lipinski C. (2006). Why Repurposing Works and How to Pick a Winning Drug While Avoiding Failures.

[13] Shoichet B.K. (2006). Screening in a spirit haunted world. Drug Discov. Today.

[14] Jarvis R.A., Patrick E.A. (1973). Clustering Using a Similarity Measure Based on Shared Nearest Neighbors *IEEE*. Trans. Comput..

[15] Brown R.D., Martin Y.C. (1996). Use of Structure-Activity Data to Compare Structure-Based Clustering Methods and Descriptors for Use in Compound Selection. J. Chem. Inf. Comput. Sci..

[16] Barnard J.M., Downs G.M. (1997). Chemical Fragment Generation and Clustering Software. J. Chem. Inf. Comput. Sci..

[17] Durant J.L., Leland B.A., Henry D.R., Nourse J.G. (2002). Reoptimization of MDL Keys for Use in Drug Discovery. J. Chem. Inf. Comput. Sci..

[18] Xu J. (2002). A New Approach to finding natural chemical structure classes. J. Med. Chem..

[19] Daylight Chemical Information Systems, Inc. Home Page.

[20] Jarvis R.A., Patrick E.A. (1973). The Jarvis-Patrick algorithm – clustering, using a similarity measure based on nearest neighbors. IEEE Trans. Comput..

